# Effects of specific nutrients on immune modulation in patients with gastrectomy

**DOI:** 10.1002/ags3.12299

**Published:** 2019-12-09

**Authors:** Jin‐Ming Wu, Ming‐Tsan Lin

**Affiliations:** ^1^ Department of Surgery National Taiwan University Hospital and National Taiwan University College of Medicine Taipei Taiwan

**Keywords:** arginine, gastrectomy, glutamine, immunonutrition, n‐3 fatty acids

## Abstract

Gastric cancer (GC) is one of the most prevalent and lethal malignant neoplasms worldwide. The main treatment for GC is gastrectomy, which generally causes considerable metabolic stress to patients. To modulate cell function, maintain homeostasis of the immune response, reduce postoperative complications, and obtain favorable outcomes, physicians prescribe specific nutrients with immunomodulatory properties as supplementation to enteral or parenteral formulas, indicating immunonutrition. In the formulas, among the immunonutrients, glutamine, arginine, and n‐3 polyunsaturated fatty acids are the most commonly used either alone or in combination. The present review summarizes and focuses on the evidence obtained from clinical trials and animal studies supporting the role of immunonutrients supplemented enterally or parenterally in total or subtotal gastrectomy. In addition, this review describes the possible molecular mechanisms underlying the protective action of these immunonutrients, which may contribute to therapeutic approaches to improve postoperative outcomes of gastrectomy. Combination of conventional therapy with immunonutrition seems to be a useful strategy to achieve synergistic effects in the treatment of GC patients.

## Graphical abstract

Gastric cancer is one of the most prevalent and lethal malignant gastrointestinal cancer, and curative gastrectomy is the main intervention to provide long‐term survival. Although gastrectomy is beneficial for the oncological outcomes, it causes considerable metabolic stress to relatively immunocompromised patients due to malnutrition and advanced cancer. The immunonutrients play an important role in restoring the function of immune system and improving the surgical outcomes.

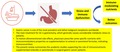

## 1 INTRODUCTION

Gastric cancer (GC) is one of the most prevalent malignancies causing mortality worldwide. It remains among the top 10 causes of cancer‐related death for both genders in many Asian countries. The primary therapeutic method for resectable GC is gastrectomy, which generally causes massive metabolic stress to patients. Patients undergoing major surgery may have an inflammatory reaction and postoperative immunosuppression, making them highly susceptible to infection.^1^ Furthermore, radical cancer surgery may result in an intestinal hypodynamic state and impair the secretion of intestinal enzymes. These conditions may compromise early recovery of the gastrointestinal tract. Most patients who undergo gastrectomy experience preoperative protein‐energy malnutrition, and adequate postoperative oral intake is achieved late. Thus, artificial nutritional support is essential for these patients. According to the consensus guidelines for enhanced recovery after gastrectomy and as recommended by the Enhanced Recovery after Surgery Society, malnourished patients should be optimized with oral supplements or enteral nutrition (EN) preoperatively. Patients who are clearly malnourished or those who cannot meet 60% of the daily requirement by postoperative day 6 should be given individualized nutritional support.^2^ For all postoperative patients who have a functioning gastrointestinal (GI) tract but cannot tolerate oral intake, standard EN is generally recommended because it is less expensive, safer, and more physiological to maintain the barrier function of the intestines than any artificial nutritional support. Meta‐analysis reports suggest that EN is conducive to a lower infection rate and a shorter hospital stay than parenteral nutrition (PN) in GI surgical patients.^3^, ^4^ However, if patients cannot receive adequate EN as a result of GI incompetency such as ileus, bowel ischemia, severe GI bleeding, or critical illness with poor enteral tolerance, then PN is required. In our previous study, we found that compared with patients without nutritional support, morbidity and mortality of malnourished gastrectomy patients with peri‐ or postoperative total parenteral nutrition (TPN) significantly decreased.^5^

To reduce postoperative complications and achieve immune homeostasis through regulating cell function, physicians introduce enteral or parenteral formulas supplemented with nutrients having immunomodulatory properties. This type of supplementation is termed immunonutrition. Commonly in the formulas, some of the immunonutrients, such as glutamine (GLN), arginine (Arg), n‐3 polyunsaturated fatty acids (PUFA), and nucleotides, are supplemented alone or in combination. Previous meta‐analyses found that immunonutrition reduces overall postoperative complications and shortens hospital stay in adult patients with major abdominal surgery.^6^, ^7^ According to a meta‐analysis specifically for total gastrectomy patients with GC, enteral immunonutrition improves cellular immunity, modulates inflammatory response, and reduces postoperative complications.^8^ Previous studies using immunonutritional regimens were conducted using either one specific nutrient or in different combinations of nutritional components. In the present review, we explored and summarized the evidence obtained from clinical trials and animal studies supporting the role of immunonutrients supplemented enterally or parenterally in total or subtotal gastrectomy. This review also describes the possible molecular mechanisms underlying the protective action of these immunonutrients, which may contribute to the therapeutic approaches to improve postoperative outcomes of gastrectomy.

## 2 NUTRITIONAL SUPPORT IN PATIENTS WITH GC RECEIVING GASTRECTOMY

### 2.1 Effects of specific nutrient combination on gastrectomy

Although immunonutrition has agreeably favorable effects, different time points of intervention may influence the outcomes after gastrectomy. In a previous study, an enteral immune‐enhancing formula enriched with Arg, n‐3 fatty acids (FA), and ribonucleic acid (RNA), named “Impact (Novartis Nutrition, Bern, Switzerland),” was given to patients with GC in two distinct time periods; then, the potential benefits of perioperative versus postoperative treatments on host defense and protein metabolism were evaluated. Compared with postoperative dosage, perioperative supplementation of immunonutrition had more metabolic advantages in modulating postoperative immune and inflammatory responses in gastrectomy patients with GC.^9^ However, early postoperative EN with Impact supplementation increased collagen synthesis, thereby promoting wound healing^10^ and improving the clinical and immunological outcomes of these patients.^11^ Okamoto et al^12^ found that preoperative EN with Impact supplementation maintained CD4^+^ T‐cell levels, shortened the duration of systemic inflammatory response syndrome, and decreased the incidence of postoperative infectious complications in patients with GC. In another study, Impact was included in a normal diet for 5 consecutive days before gastrectomy; however, preoperative enteral immunonutrition did not show any benefits in the clinical outcomes of patients with GC.^13^ Meanwhile, a prospective randomized clinical trial showed that routine postoperative immunonutrition given enterally or parenterally had no advantages in well‐nourished patients undergoing elective gastrectomy or pancreaticoduodenectomy.^14^ Peker et al^15^ investigated the effects of immunonutrition on infiltrative lymphocytes and angiogenesis in resected gastric adenocarcinoma tissues. They found that compared with the samples obtained from patients with standard nutrition, the samples from patients consuming Impact for 7 preoperative days resulted in a lower CD4^+^/CD8^+^ ratio and in a higher CD105 level. The low CD4^+^/CD8^+^ ratio in tumor‐infiltrating lymphocytes could be an indicator that tumor progression has been prevented. However, CD105 is a marker of microvascular intensity and is used to indicate neovascularity. The abovementioned findings indicated that preoperative immunonutrition regulates the balance between CD4^+^ and CD8^+^ T cells but its prolonged use increases tumor angiogenesis.^16^

In a clinical trial, malnourished patients were given preoperative PN for 2 weeks initially. Then, gastrectomy or pancreatectomy patients were randomly assigned to either a postoperative immunomodulating diet or a standard oligopeptide diet. Immunomodulating diet contains n‐3 FA, GLN, and Arg as immunonutrients. Consequently, postoperative immunonutrition reduced the overall morbidity and mortality in malnourished surgical patients with cancer.^17^ A study carried out by Liu et al^18^ used the standard enteral formula enriched with GLN and Arg for 7 days postoperatively in total gastrectomy patients with GC. They found that serum albumin, proalbumin, and transferrin levels, as well as CD4^+^ T cells, natural killer cell percentages, and immunoglobin levels were elevated prominently. Hence, postoperative immunonutrition improved the patients’ nutritional status and immune function after gastrectomy. A recent study evaluated the effect of immunonutrition on immunomodulatory and anti‐inflammatory functions in gastrectomy patients with GC. As a result, the formula enriched with Arg, GLN, n‐3 FA, and nucleotide contributed to high levels of CD4^+^ and CD3^+^ T cells and immunoglobin count, but the levels of inflammatory mediators were low. Thus, postoperative immunonutrition improved immune function and attenuated the inflammatory response in gastrectomy patients with GC.^19^ A meta‐analysis concluded that among the immunonutrients used for enteral immunonutrition, namely, Arg, GLN, n‐3 FA, and RNA, RNA and n‐3 FA are the most effective in reducing infectious complications and length of hospital stay in patients with GC suffering from gastrectomy.^20^ In a rodent sarcopenia model induced by total gastrectomy, Haba et al^21^ investigated the effect of oral branched‐chain amino acids (BCAA) and/or GLN supplementation on skeletal muscle atrophy after surgery. They found that combined use of BCAA and GLN was more effective in inhibiting muscle atrophy than providing BCAA or GLN alone. Considering that several immunonutrients are combined in enteral immunonutrition formulas, the influence of each immunonutrient on gastrectomy cannot be confirmed. Therefore, we summarized the studies that used the most commonly used immunonutrients, and we describe the roles of these immunonutrients on gastrectomy.

### 2.2 Effects of FA on gastrectomy

Fish oils contain rich sources of n‐3 FA, especially eicosapentaenoic acid (EPA) and docosahexaenoic acid (DHA). Numerous clinical trials have shown that fish oil supplementation has anti‐inflammatory and immunomodulatory properties.^22^, ^23^ EPA‐ and DHA‐acquired metabolites are associated with a decrease in proinflammatory mediators, and their ability to reduce prostaglandin E_2_ production may modulate the release of T helper cell type 2 (Th2) cytokines and elicit highly balanced T‐cell subsets during metabolic stress.^24^, ^25^

A randomized clinical trial compared n‐3 FA‐supplemented EN with a standard formula for 7 days before and after operation in patients with subtotal esophagectomy or total gastrectomy. Consequently, perioperative enteral immunonutrition with n‐3 FA did not affect the clinical outcomes of esophagogastrectomy patients.^26^ Moreover, Ida et al investigated the addition of EPA‐enriched nutrition to a standard diet in patients with GC undergoing total gastrectomy. Oral supplementation with 2.2 g EPA was provided for 7 preoperative days and 21 postoperative days. Compared with the standard diet, an EPA‐enriched oral diet did not influence surgical morbidity and body weight loss after total gastrectomy.^27^

With regard to PN, Makay et al^28^ evaluated the effect of postoperative n‐3 FA dosage on cellular hypoperfusion associated with major gastric surgery. They found that PN with n‐3 FA supplementation did not have a significant effect on lactate clearance and cellular hypoperfusion after major gastrectomy. In a prospective, randomized controlled trial, patients with GC were treated with n‐3 FA‐rich fish oil fat emulsion or received soybean oil by PN after surgery. Although mean plasma lactate concentration in patients with n‐3 FA was higher, the difference was not statistically significant. Further, no difference was observed in the changes of liver aand renal functions. Meanwhile, PN based on n‐3 FA‐rich fish oil fat emulsion alleviated the inflammatory reaction and reduced inflammatory complications after gastrectomy.^29^ SMOFlipid (Fresenius Kabi, Bad Homburg, Germany) is an i.v. lipid emulsion with a physical mixture of medium‐chain triglycerides (MCT), soybean oil, olive oil, and fish oil. We conducted a study to compare the efficacy of this lipid emulsion with that of MCT/long‐chain triglyceride (LCT) in adult patients undergoing GI surgery. Despite the clinical outcomes and inflammatory response being comparable between groups, SMOFlipid had a better triglyceride‐lowering effect. Therefore, SMOFlipid may have potential benefits in patients with limited triglyceride elimination capacity.^30^

Some basic animal and cell studies were conducted to investigate the influences of fish oil on gastrectomy. We previously evaluated the effect of n‐3 FA‐containing PN on immune functions, including leukocyte adhesion molecule expression, circulating lymphocyte subpopulation, intracellular cytokine, and phagocytic activity in rats receiving total gastrectomy. Results showed that parenterally infused fish oil did not lead to immunosuppression. In addition, giving fish oil resulted in a higher CD4^+^ T‐cell proportion and CD4^+^/CD8^+^ ratio, enhanced the peritoneal phagocytic activity of macrophages, and reduced leukocyte adhesion molecule expression in rats with total gastrectomy.^31^ An in vitro study carried out in our laboratory also showed that EPA and DHA inhibited macrophage‐induced GC cell migration, implying the beneficial effects of EPA/DHA in GC treatment.^32^ Fat emulsions containing LCT and MCT are commonly used in PN. We previously investigated the effects of pre‐infusion with TPN using MCT versus LCT emulsion on inflammatory mediators, hepatic lipids, and antioxidant capacity in rats receiving gastrectomy. Rats were inserted with an internal jugular catheter individually and then received TPN for 5 days. Thereafter, they underwent partial gastrectomy and were killed 24 hours postoperatively. We found that preoperative infusion with MCT/LCT improved hepatic lipid metabolism and reduced oxidative stress after gastrectomy.^33^ This aforementioned emulsion comprises structured triacylglycerols (TG) with medium‐ and long‐chain FA randomly distributed within a single TG molecule. This type of fat emulsion is clinically advantageous over LCT and the physical mixture of MCT/LCT emulsions because structured MCT/LCT are cleared faster from the blood and improve nitrogen balance in moderately catabolic patients.^34^, ^35^ Compared with the LCT and MCT/LCT groups, rats given structured MCT/LCT had lower plasma lipid levels and leukocyte integrin expression after total gastrectomy.^36^

### 2.3 Effects of GLN on gastrectomy

Glutamine is the most abundant amino acid in mammalian plasma and intracellular pools. Many important physiological functions depend directly or indirectly on GLN metabolism. GLN is a shuttle for nitrogen transport, an antioxidant precursor, a source of carbon and nitrogen, and an energy source for rapidly proliferating cells.^37^ GLN, as a nonessential amino acid, can be synthesized in virtually all tissues of the body. However, under certain catabolic conditions, such as trauma, surgery, or infection, metabolic demands increase, and the ability of GLN production is impaired. GLN obtained from dietary intake and endogenous synthesis becomes insufficient to meet the GLN requirement of the body. GLN may become essential, and additional supplementation is necessary during certain catabolic states.^38^ GLN has elicited considerable attention for its therapeutic role in disease treatment. The beneficial effects of providing exogenous GLN supplementation for metabolically stressful conditions have been extensively studied with positive effects of increasing nitrogen retention, preserving intestinal mucosa integrity and intestinal permeability, and modulating immunological function.^38^, ^39^

For evaluating the clinical impact of GLN on postoperative outcomes, GLN‐supplemented PN was given during the postoperative period for gastrectomy patients with GC. Consequently, GLN supplementation reduced the rate of infectious complications, improved immune function, and shortened hospital stay.^40^ A prospective randomized trial investigated the effect of GLN on postoperative immunosuppression in patients with surgical interventions on esophagus or stomach. Compared with that in the control group without GLN, parenteral GLN or n‐3 FA supplementation reversed postoperative immunosuppression, as analyzed by the percentages of T‐lymphocyte subsets and human leukocyte antigen‐DR isotype (HLA‐DR) expression on monocytes.^41^ Spittler et al^42^ investigated postoperative glycyl‐GLN infusion on HLA‐DR expression on monocytes in major abdominal surgery patients. HLA‐DR is a gene product of the major histocompatibility complex (MHC) class II antigen. HLA‐DR surface antigen expression on monocytes is reduced by surgery and inflammation and is associated with an increased susceptibility to infectious complications. Amino acid solution containing 35 g GLN was infused immediately after surgery and continuously for the next 48 hours. Compared with that in controls without GLN supplementation, giving GLN preserved HLA‐DR expression on monocytes, thereby possibly preventing infectious postoperative complications.^43^ Moreover, Mochiki et al^44^ investigated postoperative treatment with GLN on GI motor activity in partial distal gastrectomy of patients with GC. Compared with the control, patients receiving oral GLN supplementation (3 g/d) had greater duodenal motor activity and interdigestive migrating motor contractions. Thus, GLN could act as a motility‐recovery agent after gastrectomy.

As for animal studies, rats were infused with GLN‐containing PN before and after gastrectomy to investigate the efficacy of GLN on surgery. As a result, TPN supplemented with GLN improved nitrogen balance and enhanced abdominal macrophage phagocytic activity in rats with partial gastrectomy.^45^ In a previous study, we gave GLN parenterally to investigate the role of GLN on cellular adhesion molecule and inflammatory cell recruitment in rats with total gastrectomy. We found that GLN‐enriched PN attenuated leukocyte integrin expression and elicited a more rapid immune response to injury after gastrectomy.^46^

### 2.4 Effects of Arg on gastrectomy

Arginine is a nonessential amino acid that serves as the precursor of various metabolites, such as nitric oxide (NO), citrulline, creatine, ornithine, urea, and polyamines.^47^ Arg stimulates anabolic hormone release and improves nitrogen balance.^48^ It also shows immunoregulatory characteristics^49^ and can attenuate an inflammatory response in stressful conditions.^50^ Conversely, Arg deficiency or unavailability inhibits T‐lymphocyte proliferation, resulting in diverse impairments in the immune system and response.^51^ The resulting immune dysfunction is associated with an increased risk of infection in critically ill patients.^52^ Arg is considered a conditionally essential amino acid for catabolic patients.^53^, ^54^ However, Arg supplementation remains controversial, especially for patients with sepsis. Although meta‐analysis studies found that Arg supplementation has no effect on infectious complications and may deteriorate outcomes in critically ill patients, Arg supplementation is associated with reduced infectious complication rates and a short length of hospital stay with no adverse effects on mortality in patients receiving elective surgery.^55^ Recently, in human studies, Arg has been found to decrease clinical infections, postoperative hospital stay, intra‐abdominal abscess and anastomotic leak, and mortality.^53^, ^56^ A review reported that the dosage of Arg available in commercial formulations is nontoxic and that parenteral delivery of Arg is also safe at clinical levels. Therefore, Arg should be considered for inclusion in immunonutrient combinations in patients who have suffered trauma and those in acute surgical settings.^57^

Although Arg is a component commonly included in the immunonutrition formula, exclusive use of Arg in clinical trials of major abdominal surgery is rare. To date, Arg supplementation in total gastrectomy has been seldom studied. Kamocki et al^58^ investigated the effect of perioperative immunonutrition on platelet phagocytic activity in gastrectomy or laparotomy patients with advanced GC. They found that the phagocytic activity of platelets in advanced GC patients is significantly impaired and perioperative immunonutrition can partially restore platelet phagocytic activity. Compared with perioperative immunonutrition with GLN and n‐3 FA, oral Arg supplementation along with adjunct nutritional therapy significantly increased the fraction of phagocytizing platelets and improved the phagocytic index of thrombocytes. In an animal study, Yeh et al^59^ evaluated Arg‐containing PN on abdominal phagocytic activity in a model of partial gastrectomy. They found that parenteral supplementation of Arg enhanced phagocytic activity and reduced the production of inflammatory mediators after injury. Studies showing the effectiveness of enteral and parenteral immunonutrition on gastrectomy are summarized in Tables 1 and 2.

**Table 1:** Studies on enteral immunonutrition in gastrectomy

Reference	Immunonutrients	Time point	Outcomes
Braga et al^9^	Arg, n‐3 FA, and RNA	Perioperation	Advantages in modulating postoperative immune and inflammatory responses
Farreras et al^10^	Arg, n‐3 FA, and RNA	Postoperation	Increases collagen synthesis and improves wound healing
Okamoto et al^12^	Arg, n‐3 FA, and RNA	Preoperation	Maintains CD4^+^ T‐cell levels, shortens duration of systemic inflammatory response syndrome, and decreases the incidence of postoperative infectious complications
Klek et al^17^	n‐3 FA, GLN, and Arg	Postoperation	Reduces overall morbidity and mortality in malnourished patients
Fujitani et al^13^	Arg, n‐3 FA, and RNA	Preoperation	No benefits in clinical outcomes
Liu et al^18^	GLN and Arg	Postoperation	Improves nutritional status and immune function
Sultan et al^26^	n‐3 FA	Perioperation	No benefits in clinical outcomes
Marano et al^11^	Arg, n‐3 FA, and RNA	Postoperation	Improves clinical and immunological outcomes
Kamocki et al^58^	Arg	Perioperation	Increases the fraction of phagocytizing platelets and improves the phagocytic index of thrombocytes
Peker et al^15^	Arg, n‐3 FA, and RNA	Preoperation	Regulates the balance between CD4 and CD8 T cells but increases tumor angiogenesis with prolonged use
Ida et al^27^	EPA	Perioperation	No benefits in surgical morbidity and body weight loss
Li et al^19^	Arg, GLN, n‐3 FA, and nucleotide	Postoperation	Improves immune function and attenuates inflammatory response
Animal study			
Haba et al^21^	BCAA and GLN	Postoperation	Inhibits muscle atrophy after surgery

**Table 2:** Studies on parenteral immunonutrition in gastrectomy

Reference	Immunonutrients	Time point	Outcomes
Jacobi et al^41^	GLN or n‐3 FA	Postoperation	Reverses postoperative immunosuppression as analyzed by percentages of T‐lymphocyte subsets and HLA‐DR expression on monocytes
Klek et al^40^	GLN	Postoperation	Reduces the rate of infectious complications, improves immune function, and shortens hospital stay
Klek et al^14^	GLN and n‐3FA	Postoperation	No benefits
Makay et al^28^	n‐3FA	Postoperation	No benefits in cellular hypoperfusion and lactate clearance
Wei et al^29^	n‐3FA	Postoperation	Alleviates the inflammatory reaction and reduces inflammatory complications
Wu et al^30^	Mixtures of n‐3, n‐6, n‐9 FA and MCT	Postoperation	Have a favorable triglyceride‐lowering effect
Animal studies			
Spittler et al^42^	GLN	Postoperation	Preserves HLA‐DR expression on monocytes, preventing infectious complications after surgery
Lin et al^33^	MCT/LCT	Preoperation	Improves liver lipid metabolism and reduces oxidative stress
Yeh et al^59^	Arg	Postoperation	Enhances phagocytic activity and reduces production of inflammatory mediators at site of injury
Lin et al^31^	n‐3 FA	Postoperation	Increases CD4^+^ T‐cell proportion and CD4^+^/CD8^+^ ratio, enhances peritoneal macrophage phagocytic activity, and reduces leukocyte adhesion molecule expression
Lin et al^36^	structured MCT/LCT	Postoperation	Decreases plasma lipid levels and leukocyte integrin expression
Lee et al^45^	GLN	Perioperation	Improves nitrogen balance, enhances abdominal macrophage phagocytic activity
Lin et al^46^	GLN	Postoperation	Attenuates leukocyte integrin expression and elicits a highly rapid immune response to injury
Mochiki et al^44^	GLN	Postoperation	Increases duodenum motor activities and interdigestive migrating motor contractions

## 3 CONCLUSION

In the present review, although we narrowed down the populations of patients to gastrectomy, the findings among the studies have inconsistencies. The fundamental and nutritional status of the recruited patients, the stage and severity of GC, the methods of operation, the intervention dosage, and the time of measuring outcomes are all confounding variables that make each clinical trial different from others. Meanwhile, data derived from animal studies cannot be extrapolated to humans, and studies with a small sample size may have a low statistical power that requires cautious interpretation. However, most studies reviewed in this report suggested that immunonutrients that are provided either alone or in combination have favorable effects in improving patient outcomes, except that the exclusive use of arginine is rare and needs to be confirmed in gastrectomy patients with GC. Conventional therapy in combination with immunonutrition pre‐ and/or postoperatively may be an important strategy to achieve favorable synergistic effects in the management of GC patients.

## DISCLOSURE

Conflicts of Interest: Authors declare no conflicts of interest for this article.
